# Phylogenomic resolution of lampreys reveals the recent evolution of an ancient vertebrate lineage

**DOI:** 10.1098/rspb.2024.2101

**Published:** 2025-01-08

**Authors:** Lily C. Hughes, Devin D. Bloom, Kyle R. Piller, Nicholas Lang, Richard L. Mayden

**Affiliations:** ^1^ Department of Marine, Earth, and Atmospheric Sciences, North Carolina State University, Raleigh, NC 27606, USA; ^2^ North Carolina Museum of Natural Sciences, Raleigh, NC 27601, USA; ^3^ Department of Biological Sciences, Western Michigan University, 1903 W Michigan Avenue, Kalamazoo, MI 49008, USA; ^4^ School of the Environment, Geography, and Sustainability, Western Michigan University, 1903 W Michigan Avenue, Kalamazoo, MI 49008, USA; ^5^ Department of Biological Sciences, Southeastern Louisiana University, Hammond, LA 70402, USA; ^6^ Science Department, Lane Tech College Prep High School, Chicago, IL 60618, USA; ^7^ Department of Biology, Saint Louis University, St Louis, MO 63103, USA

**Keywords:** Petromyzontiformes, biogeography, jawless fishes, divergence time estimation, diversification, fossilized birth–death model

## Abstract

Jawless vertebrates once dominated Palaeozoic waters, but just two lineages have persisted to the present day: lampreys and hagfishes. Living lampreys are a relatively small clade, with just over 50 species described, but knowledge of their evolutionary relationships has always been based on either a few mitochondrial genes or a small number of taxa. Biogeographers have noted the disjunct antitropical distribution of living lamprey families. Here, we present a comprehensive phylogenomic analysis of living and fossil lampreys, sampling 36 species with phylogenomic data and 46 in total with genetic data. We present new divergence time estimates based on comprehensive nuclear data and analysis of their diversification dynamics. Our analysis indicates a central role for extreme global warming during the Late Cretaceous Cenomanian–Turonian Boundary Event as a likely cause for the antitropical distribution of living lampreys, and a notable increase in lineage diversification in Northern Hemisphere lampreys during the Miocene corresponding with a period of global cooling.

## 1. Background

The lampreys (Petromyzontiformes) are one of two ancient surviving lineages of jawless vertebrates, with an evolutionary history stretching back hundreds of millions of years. Lampreys are celebrated for their cultural and ecological importance [1], and as one of the earliest vertebrate lineages, they reside in a key phylogenetic position that has been challenging to resolve [2]. They have diverse life history strategies, but all species spend most of their lives buried in the substrate during their larval stage, called ammocoetes. As adults many species feed on other fish as ectoparasites with their oral disc, such as the parasitic sea lamprey (*Petromyzon marinus*), whose invasion utterly devastated the Great Lakes ecosystem over the last century [3]. However, more than half of all lamprey species are non-parasitic, and many native lamprey species provide key ecosystem functions and are of conservation concern [4]. Despite their ecological and evolutionary significance, our understanding of lamprey phylogeny is limited, primarily informed by one to four genes [2,5–8], a small number of morphological characters [9] or limited taxonomic sampling [10]. Poor understanding of lamprey phylogeny in turn hampers our understanding of their fascinating and unique biology.

Extant lampreys have an antitropical distribution, with the depauperate families Geotriidae (2 spp.) and Mordaciidae (3 spp.) in the Southern Hemisphere, and the more speciose Petromyzontidae (47 spp.) in the Northern Hemisphere. The origin of this disjunct distribution is unclear, and conflicting hypotheses have been proposed. Unfortunately, lampreys and hagfishes both have a sparse fossil record, with the first definitive stem lamprey with a recognizably modern body plan from the late Devonian [11]. This is followed by a small number of fossils from the Carboniferous [12,13], two recently described from the Jurassic [14], and one in the Early Cretaceous [15]. Stem lampreys from the Carboniferous Mazon Creek Formation indicate that lampreys were not always absent from equatorial environments [16]. These fossil representatives are critical for understanding vertebrate evolution but provide few data points for the past distribution of lampreys.

Given their ancient origins and a classic Gondawanan and Laurasian distribution, the break-up of Pangea is an appealing and intuitive hypothesis to explain modern-day lamprey distribution [6]. This hypothesis rests on two key pieces of evidence: monophyly of Northern and Southern Hemisphere lampreys and an Early Jurassic divergence between Northern and Southern Hemisphere lineages. Morphological data have been unable to convincingly resolve lamprey relationships [2,9] and some molecular analyses have recovered the Southern Hemisphere families as paraphyletic [14], though recent genome-scale data have supported Southern Hemisphere monophyly [10].

However, vicariance via tectonic drift is not the only possible explanation for disjunct antitropical distributions. Physiological limitations to tolerating higher temperatures can exclude taxa from tropical habitats, and lampreys in their ammocoete larval form do not tolerate high temperatures [17–19]. Ammocoetes are a derived feature of lamprey evolution, and Palaeozoic lampreys inhabiting equatorial environments did not possess this developmental stage [20]. Climate oscillations may allow temperate-adapted taxa to disperse through equatorial provinces during cooler periods [21]. A recent study that did not resolve Southern Hemisphere families as monophyletic obtained a younger 78 Ma age estimate for crown Petromyzontiformes [14]. They hypothesized that lamprey antitropicality arose when lampreys dispersed out of the Southern Hemisphere during a cooler period between the extreme heat of the Cenomanian–Turonian Boundary Event (CTBE, maximum = *ca* 93 Ma) and the Palaeocene–Eocene Thermal Maximum (PETM, maximum = *ca* 56 Ma) [14].

Reconciling molecular age estimates and a well resolved phylogeny for crown Petromyzontiformes are critical for differentiating between these hypotheses. To date, divergence time estimation for lampreys has relied exclusively on just three mitochondrial genes, potentially misleading age estimates [22]. Phylogenomic methods that can capture and sequence hundreds to thousands of genes for non-model species are now commonplace [23] and can provide a more comprehensive dataset to test these hypotheses and estimate divergence times with nuclear DNA.

We present a phylogenomic analysis for extant lamprey species that resolves phylogenetic relationships and clarifies taxonomic uncertainties. By combining two morphological matrices with extant and extinct taxa and genome-scale data, we provide a total-evidence dating analysis to explore divergence times among major lamprey lineages, including testing for the hypothesis that the breakup of the supercontinent Pangea explains the antitropical distribution of extant lampreys. Finally, we explore diversification rates among extant lampreys to determine the macroevolutionary dynamics that underlie the highly disparate diversity patterns among lamprey clades.

## 2. Material and methods

### (a) Taxonomic sampling

We obtained 81 tissue or DNA samples from 36 of the 48 currently recognized lamprey species and one undescribed species [24], plus five hagfish outgroup samples. We also incorporated *Geotria autralis*, *Mordacia mordax* and a hagfish outgroup sample from published transcriptomes [25]. To provide a complete lamprey tree, we used *cytochrome b* mitochondrial sequences (*cytb*) from GenBank to add 10 species not included in our phylogenomic dataset (see below). Specimen information and Sequence Read Archive (SRA) accession numbers are listed in electronic supplementary material, table S1; *cytochrome b* accession numbers are in electronic supplementary material, table S2.

### (b) Exon probe design and sequencing

We designed new exon-capture probes from the sea lamprey (*P. marinus*) genome annotation for protein-coding genes [26]. Lampreys systematically eliminate a portion of their genome from their somatic cells, with the complete genome only found in germline cells [27]. As we were using somatic tissue from vouchered museum collections across Petromyzontiformes, searching only the somatic sea lamprey genome was more appropriate. Using HMMER3 [28], we searched a set of 1104 single-copy exons designed for actinopterygiian phylogenomics against *P. marinus* and extracted matching sequences [29,30]. Out of 1104 nuclear exons, 397 were identified in the sea lamprey genome. Probes 100 nt in length were designed and filtered for repetitive sequences, hybridization, and melting temperature by Arbor Biosciences (Ann Arbor, MI, USA) based on these reference sequences. Target capture and paired-end sequencing on an Illumina HiSeq 2500 were also performed by Arbor Biosciences. Probe sequences are available on Dryad.

### (c) Matrix assembly and phylogenomic analysis

The resulting raw Illumina reads were quality-trimmed with Trimmomatic v. 0.33 [31]. Trimmed sequencing reads were assembled by locus using a pipeline designed to assemble ray-finned fish exon capture data [26], modified with *P. marinus* reference sequences. This pipeline uses aTRAM 2.0 to iteratively assemble loci [32], using the Trinity v. 2.13 assembler [33]. Published transcriptome sequencing data were processed through this pipeline for *M. mordax* and *Geotria australis* [25]. Target-captured hagfish (Myxini) outgroups were initially assembled under the same parameters but yielded few assembled loci (<10 per sample) owing to the ancient divergence between these living jawless fish lineages. However, shotgun assembly of hagfish samples with Trinity v. 2.13 [33] and searching for the subset of exon loci with nHMMER identified 68–74 exons of our set of 397 in these hagfish samples [29]. Sequences from eight ray-finned fish genomes were incorporated as additional outgroups (*Anguilla anguilla*, *Lepisosteus oculatus*, *Danio rerio*, *Gadus morhua*, *Oryzias latipes*, *Oreochromis niloticus*, *Gasterosteus aculeatus* and *Tetraodon nigroviridis*). Exons were aligned with MACSE v. 2.03 [34] and inspected by eye. These alignments were filtered for potential contamination following a strict quality control pipeline following the steps of Arcila *et al*. [35]. This included removing any identical sequences identified with BLAST+ found across different clades, correlating constrained gene-tree and concatenated-tree branch lengths and excluding sequences in individual genes with excessively long gene-tree branches [36], and applying the TreeShrink algorithm [37]. Alignments with fewer than 10 sequences were excluded, giving a final total of 355 exons included for analysis.

We conducted a concatenated analysis in IQ-Tree v. 2.2.0, fully partitioned by gene and codon position with the best-fitting models and partitioning scheme tested with ModelFinder [38–40], and 1000 ultrafast bootstrap replicates were conducted to assess branch support [41]. Given the long branches leading to Southern Hemisphere lampreys, there is the potential for heterotachy—rate heterogeneity through time which might make these lineages prone to long-branch attraction artefacts. Thus, the data were also analysed under the GHOST model [42], which accounts for heterotachy, with a reduced dataset of one individual per species given the increased complexity of this model. For analysis under the multispecies coalescent (MSC) in ASTRAL-III [43], we first estimated individual gene trees with IQ-Tree, partitioned by codon position and the best-fitting models and partitioning scheme identified by ModelFinder, and 1000 ultrafast bootstrap replicates. We ran ASTRAL with these gene trees as input, and a second analysis where nodes in gene trees with less than 30% bootstrap support were collapsed. Using the individual gene trees, we also estimated the gene concordance factors (gCF) and likelihood-based site concordance factors (sCFL) in IQ-Tree based on our Maximum Likelihood (ML) topology [44,45].

### (d) Total evidence dating

For total evidence dating, we combined two morphological matrices containing extinct and extant cyclostomes. From a large study of vertebrate lineages containing 168 characters [2], we subsampled the variant characters for cyclostomes. We incorporated an additional 32-character lamprey-only matrix [9], with fossil codings recently added for †*Mesomyzon mengae* [6]. Overlapping characters between the two datasets were merged into a single character, and character states were re-coded starting from zero for consistency. Three five-state characters were re-coded to three states, as BEAST2 partitions morphological characters by the number of character states. These characters are noted in the NEXUS file, along with their descriptions. In total, our combined matrix contained 65 morphological characters. We analysed the matrix under a new technology search in TNT with an initial addition of 1000 sequences and 100 ratchet iterations [46]. Recently published Jurassic lampreys were not included in both datasets, and thus are not analysed here, but we discuss similarities and differences between our results.

A subset of 148 exons with reduced missing data and just one individual per species were selected for divergence time analysis. *Entosphenus folletti* was consistently found nested in *Entosphenus tridentatus*, and *Entosphenus similis* was consistently found nested in *Entosphenus minimus*. These species were excluded from this analysis. The best-fitting substitution model was selected for three partitions, defined by codon position. A total-evidence matrix combining DNA and morphology was analysed under the Fossilized Birth–Death Model (FBD) in BEAST2 under the Optimized Relaxed Clock Model (ORC) [47–50]. The uniform Origin Time (*x*_0_) prior is based on the age of the Late Devonian †*Priscomyzon riniensis*, the oldest definitive member of Petromyzontida [11]. We set a minimum age of 359 Ma, slightly older than the fossil, and a maximum age of 636.1 Ma based on the recommendation of Benton *et al*. [51]. Given that the fossil record of lampreys is very ancient but also very sparse, we set an exponentially distributed prior (mean = 0.25) on the sampling probability (*ρ*), giving higher weight to lower values. The same logic and prior distribution were used for diversification rate (*d*). Turnover (*r*) was assigned an uninformative uniform prior. The monophyly of hagfish and lamprey lineages was enforced at the root of the tree. The Cretaceous lamprey †*Mesomyzon mengae* has been phylogenetically placed as both a stem and a crown lamprey [2,6,14]; we did not constrain its placement in our tree. We executed two independent runs for at least 600 000 000 generations, with an initial 10 million generation pre-burn-in and sampling every 1000 generations. Convergence was assessed via ESS values >200 of combined runs after excluding an additional 25% burn-in fraction.

We conducted an additional BEAST2 analysis to increase taxonomic sampling to include 10 species not included in our phylogenomic dataset. We incorporated *cytochrome b* mitochondrial sequences (*cytb*) from GenBank [5,7] (electronic supplementary material, table S2) and initiated a second BEAST run with node priors based on the first nuclear DNA-only phylogenomic results. Given the high proportion of missing data for *cytb*-only taxa, we constrained their position in the tree by genus. Each of these constraints was also given a uniform prior on divergence times based on the 95% highest posterior density (HPD) intervals of the phylogenomic-scale combined BEAST runs. This was to minimize the effect of the mitochondrial loci on the overall divergence times, except where it was the only available data.

### (e) Macroevolutionary analysis of diversification rates

A sample of 10 000 trees from the posterior distribution of this run was used in downstream comparative analyses. Lineage through time (LTT) plots based on the consensus of those 10 000 posterior trees were visualized with the R packagess *ape* and *phytools* [52,53]. We simulated 1000 trees under the null expectation of a Yule Model (pure birth) and calculated the gamma statistic [54].

We explored diversification rates using BAMM v. 2.5.0 and the *BAMMtools* R package [55,56]. BAMM uses a reversible-jump Markov chain Monte Carlo (MCMC) to estimate rate heterogeneity among branches on a phylogeny. We used the speciation–extinction option to detect rate shifts along branches. We ran four chains for 10 million generations, sampling every 1000 generations. We generated priors using the ‘setBAMMpriors’ function in the *BAMMtools* package and explored the effects of priors by varying the ‘expectedNumberOfShifts’ parameter to 0.1, 1 and 10. Because we have complete taxon sampling, we could directly analyse our Maximum Clade Credibility (MCC) tree resulting from our BEAST2 analyses. Fossil taxa were pruned from the tree using the ‘drop.tip’ function in the *phytools* R package. We confirmed convergence of MCMC runs, acceptable effective sample sizes of the log-likelihood, and number of rate shifts using the ‘effectiveSize’ function in the R package *CODA* [57] and discarded 10% of the posterior sample as burn-in. We determined shift configuration with the highest posterior probability using the ‘getBestShiftConfiguation’ function in *BAMMtools*.

## 3. Results

### (a) Phylogenomic resolution

**Figure 1.**
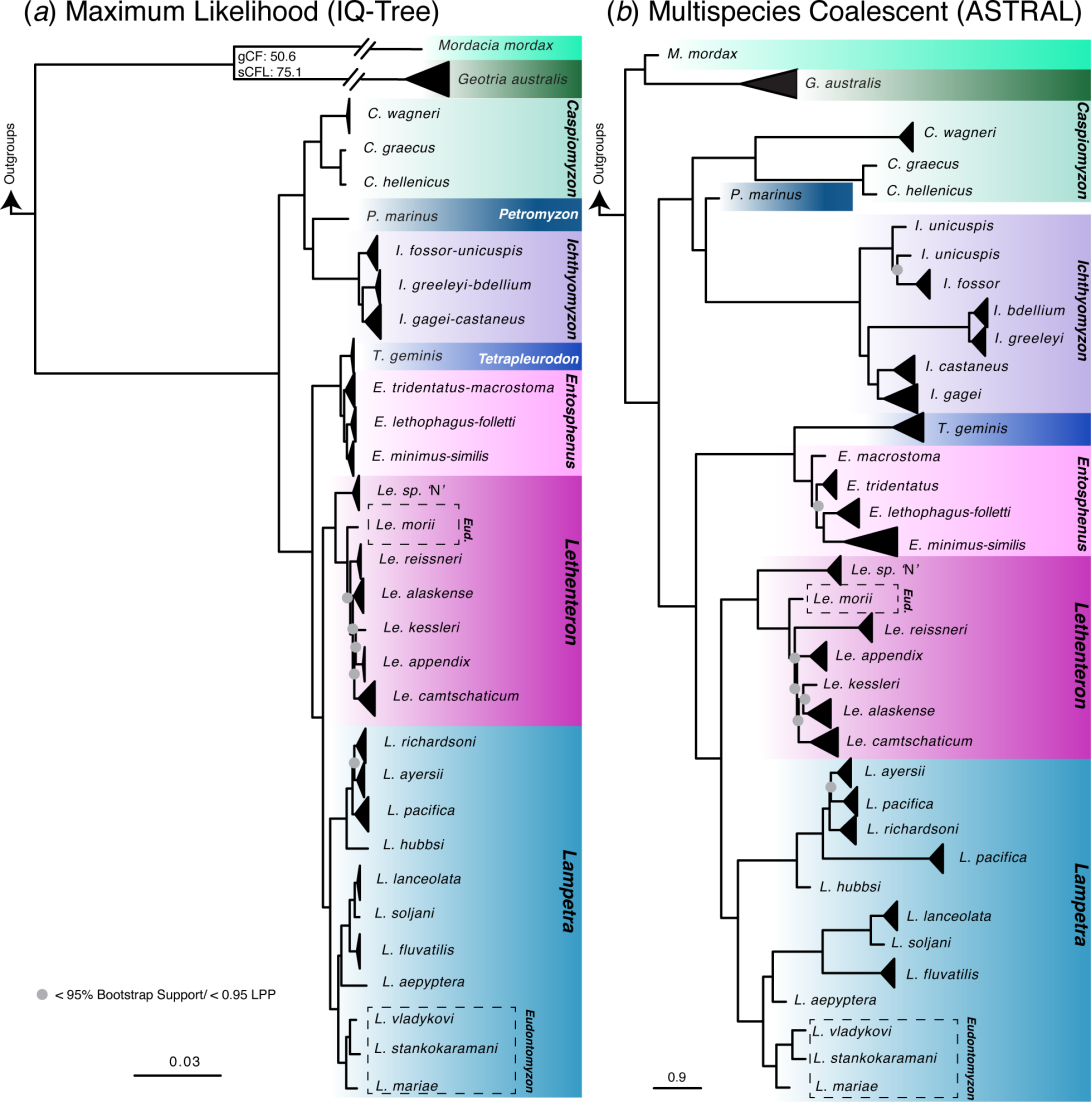
(*a*) Maximum likelihood phylogeny of Petromyzontiformes, with outgroups excluded from the figure owing to long branch lengths. Nodes without support values have >95% ultrafast bootstrap support. The gCF and sCFL values for the sister relationship between Geotriidae and Mordaciidae are shown at that node. Species pairs that are not reciprocolly monophyletic in the ML tree are shown as a collapsed clade with both species names. Taxon names surrounded by dashed boxes indicate species formerly considered in *Eudontomyzon*. (*b*) Multispecies coalescent species tree estimated in ASTRAL-III. Some species pairs are resolved as monophyletic species in ASTRAL and are shown as separate clades. Nodes with grey dots indicate local posterior probability (LPP) values <0.95; nodes without values have >0.95 support.

We obtained a strongly supported genomic phylogeny with 75% complete taxonomic sampling, with all major nodes consistent and highly supported across both concatenation (figure 1*a*; figures S1-S2) and species-tree methods (figure 1*b*; figures S3-S4). Our tree resolves the relationships among the three extant families, with Geotriidae and Mordaciidae as sister taxa in all analyses, including high support under the GHOST model incorporating heterotachy (figure S2). Concordance factor analysis returned a gCF value of 50.64, indicating that roughly 50% of gene trees supported the monophyly of Geotriidae and Mordaciidae. Lower levels of discordant trees were found in roughly equal proportions for the alternative topologies. The sCFL value was 75.1 for this node (figure 1*a*).

We present a novel resolution for the position of western North American lampreys in the genus *Lampetra* (*L. hubbsi, L. pacifica, L. ayersii* and *L. richardsonii*) as the sister to all European lampreys in the genera *Lampetra* and *Eudontomyzon*. Differences between topologies were toward the tips and limited to recent species-pairs (figure 1*a*). Non-parasitic *Ichthyomyzon gagei* samples (*n *= 6) are not monophyletic in our concatenated analyses, with parasitic *Ichthyomyzon castaneus* individuals (*n *= 3) nested within. However, individuals from these two species were reciprocally monophyletic in all ASTRAL-III analyses (figure 1*b*), suggesting a role for incomplete lineage sorting in this young species divergence. There is similar uncertainty in the relationships of individuals among other parasitic/non-parasitic species-pairs, including *Entosphenus macrostoma* and *E. tridentatus; Ichthyomyzon fossor* and *Ichthyomyzon unicuspis; Ichthyomyzon bdellium* and *Ichthyomyzon greeleyi*. Relationships among species within young lamprey genera typically had lower support, especially within *Lethenteron*. The only sampled individual of *E. folletti* was consistently nested in *Entosphenus lethophagus* samples, and the two individuals sampled from *E. similis* were consistently nested in *E. minimus* samples. These two species were dropped from downstream analysis in BEAST.

### (b) Divergence times

**Figure 2.**
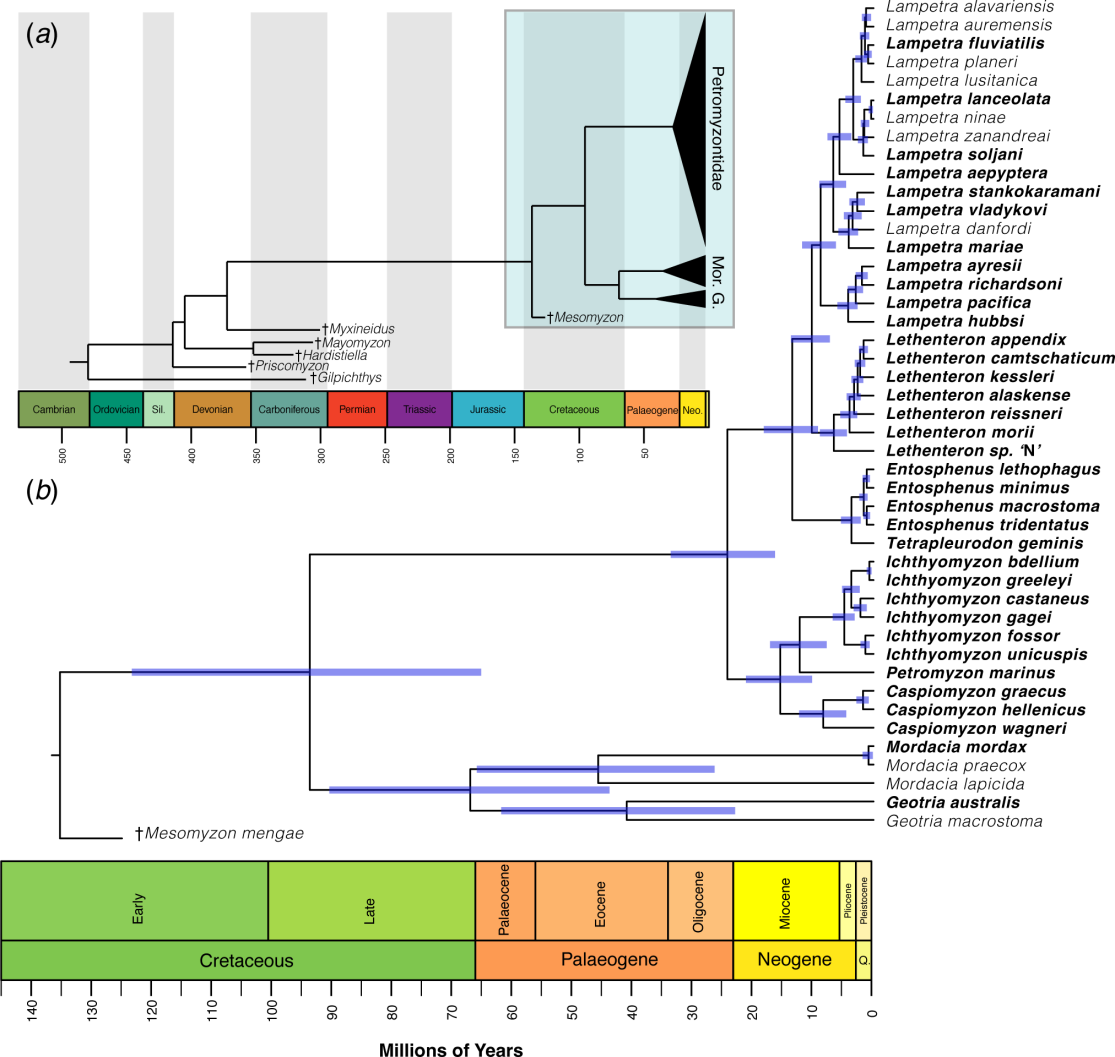
(*a*) Divergence times (Ma) of Petromyzontiformes and their stem relatives estimated in BEAST. Box indicates the region of the tree that is expanded in (*b*). Mordaciidae and Geotriidae are abbreviated as ‘Mor.’ and ‘G.’ respectively. (*b*) Divergence times of crown Petromyzontiformes in detail. Blue bars at nodes indicate the highest posterior density (HPD) intervals. Species names in bold indicate individuals sampled with phylogenomic nuclear data; all other samples are only represented with *cytochrome b* sequences.

**Figure 3.**
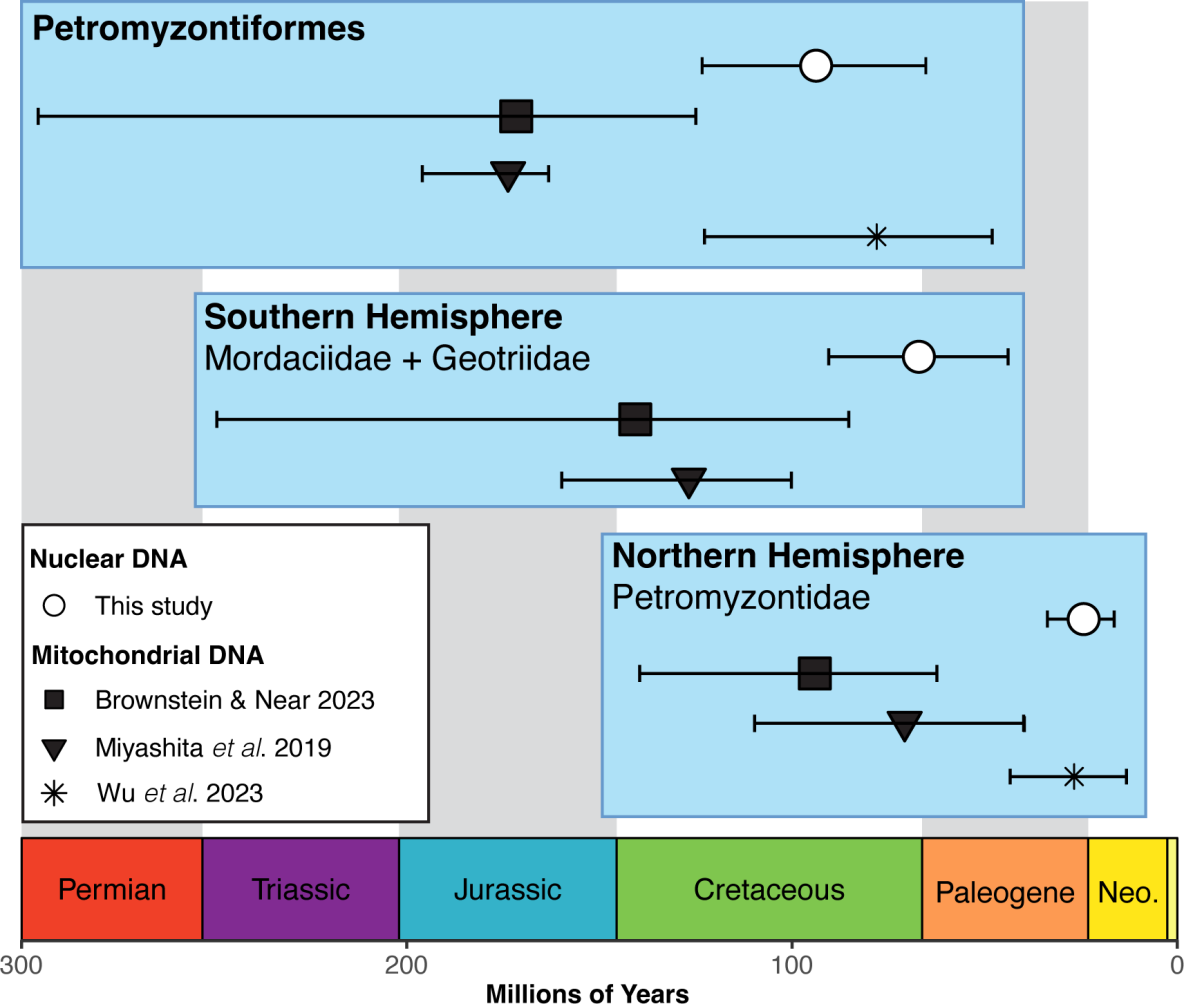
Comparison of divergence times from this study using phylogenomic-scale nuclear DNA data with three previous studies [2,6,14] that relied on mitochondrial DNA. Wu *et al*. [14] did not obtain Southern Hemisphere lampreys as a monophyletic group; thus no age is shown for that node.

The New Technology Search in TNT yielded three equally parsimonious trees with a length of 113. The strict consensus tree did not resolve relationships among several lineages, resulting in a polytomy for *Mordacia*, *Geotria*, †*Priscomyzon*, †*Myxineidus* or †*Mesomyzon* (electronic supplementary material, figure S5). Our Bayesian total-evidence analysis supported all fossil lampreys as stem lineages, including †*Mesomyzon mengae* (figure 2*a*). The topology for all fossil taxa obtained under a Bayesian search was identical to that of Miyashita *et al*. [2], but our ages are substantially younger than previous estimates (figure 3). While the total group stretches back to the Ordocivian, we estimate that crown lampreys emerged in the Cretaceous, with a mean age estimate of 93.8 Ma (figure 2*b*; HPD: 65.3–123.4 Ma). The split between the Southern Hemisphere families Mordaciidae and Geotriidae dates to a mean of 67.1 Ma (HPD: 43.9–90.5 Ma). The Northern Hemisphere lampreys are significantly younger, with an origin at the Oligocene–Miocene boundary, 24.3 Ma (HPD: 16.4–33.7 Ma), with most genera established in the Miocene or Pliocene. *Entosphenus* is the youngest genus, radiating at just 1.6 Ma in the Pleistocene.

### (c) Macroevolutionary analysis of diversification rates

**Figure 4.**
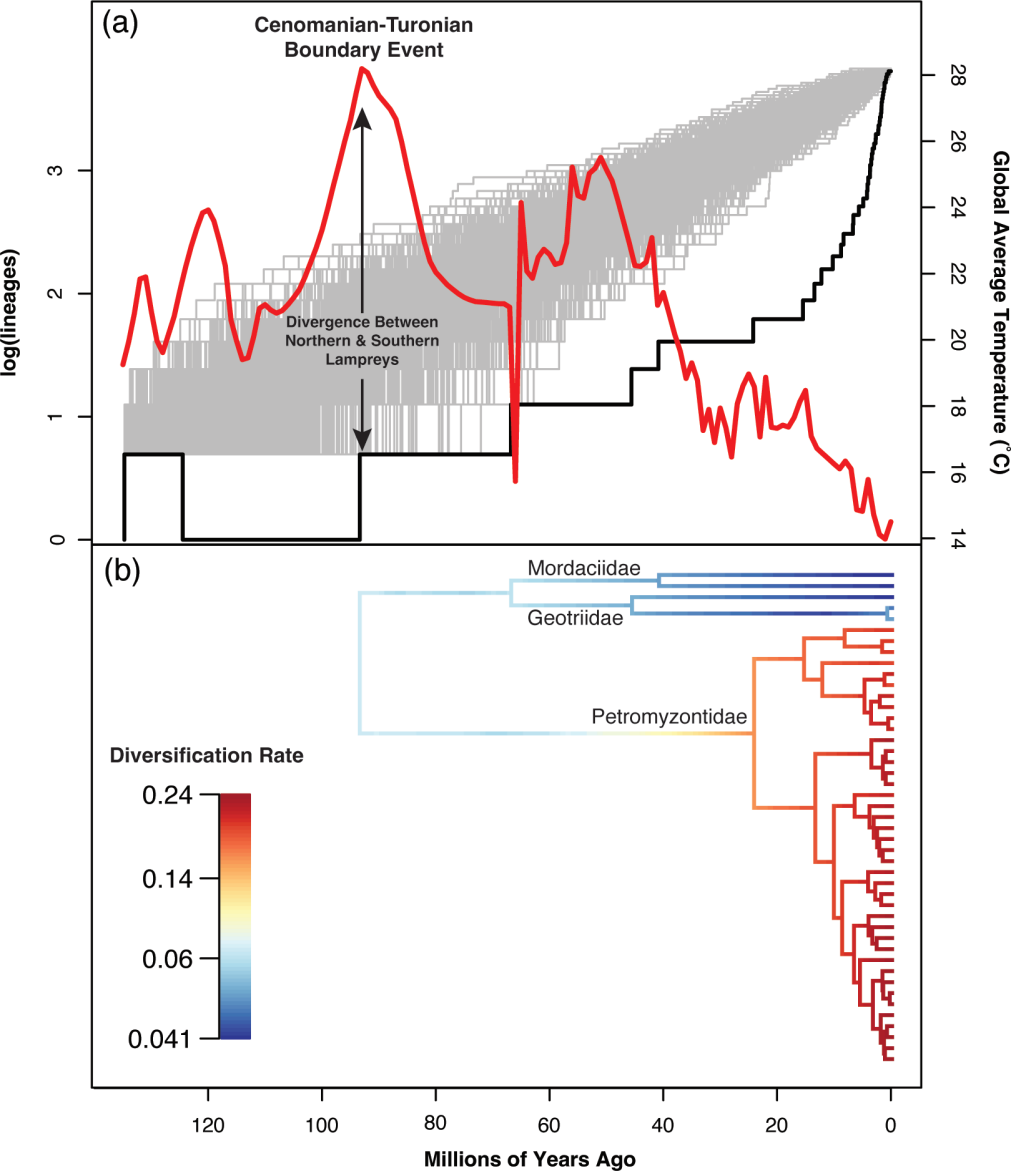
(*a*) Lineage through time (LTT) plot for Petromyzontiformes, shown with a black line over 1000 simulated trees under the Yule model shown in grey. Global average temperature is plotted in red for the same time interval [58]. The vertical black arrows indicate the split of Petromyzontidae from Geotriidae+Mordaciidae coinciding with the extreme global warming at the Cenomanian–Turonian Boundary Event. (*b*) Diversification rates (lineages per million years) across the phylogeny inferred with BAMM under the preferred model with a single rate shift along the branch subtending Petromyzontidae.

Our results rejected the null hypothesis of constant diversification rates through time (gamma = 5.4506, *p* = 0.0). Instead, we observe that living lampreys accumulated lineages slower than expected under the null model until a dramatic increase during the last 20 million years (figure 4*a*). Our diversification analysis indicated a single rate shift on the stem lineage of Petromyzontidae as the best rate configuration. Visualization of rate variation on the phylogeny shows diversification rates are relatively slow in Southern Hemisphere lampreys compared with Northern Hemisphere lampreys. Rates also appear to have accelerated over time in Northern Hemisphere lampreys, with the highest rates near the tips of the phylogeny (figure 4*b*). This pattern is confirmed by the LTT plots, which indicate accelerating rates towards the present day.

## 4. Discussion

### (a) Phylogenomics clarifies the taxonomy of living lampreys

Our results provide a well resolved backbone topology with near-complete taxonomic sampling for Petromyzontiformes. Higher-level relationships are resolved with strong support, including a sister relationship between the Southern Hemisphere families Geotriidae and Mordaciidae. A previous study based on whole-genome sequencing found high bootstrap support but low site concordance factors for this relationship [10]. However, those values were based on a parsimony calculation that can be influenced by homoplasy [45]. Using the updated likelihood calculation for this metric, we obtain a much higher site concordance factor value for our dataset.

The genus-level taxonomy of Petromyzontidae has long been contentious [59], and other recent studies have noted the need for a revision [7,10]. For the first time, we resolve the position of Pacific *Lampetra* as sister to all other Atlantic *Lampetra+Eudontomyzon*. Previous studies using only mtDNA have found this clade branching earlier in the phylogeny, rendering *Lampetra* non-monophyletic, and those authors have generally designated this group as *‘Lampetra*’ pending further investigation [5]. Given our results, we make two simple taxonomic recommendations to reconcile lamprey genera in our phylogenomic tree: (i) reclassify *Eudontomyzon morii* to *Lethenteron morii*, and (ii) sink the remaining European *Eudontomyzon* species into *Lampetra,* uniting the genus into a young, holarctic lineage. Previous authors have also suggested *Eudontomyzon* as a subgenus of *Lampetra* [59]. These recommendations are summarized in electronic supplementary material, table S3 and are used in figure 2*b*. Uncertainty remains in disentangling relationships within these extremely young lamprey genera, for which our highly conserved exon set is not well suited. These groups would benefit from denser, range-wide sampling of individuals to clarify these relationships.

Detailed, range-wide genome-scale studies are needed to determine species boundaries between parasitic and non-parasitic species-pairs. These paired species with different life histories may represent ecotypes rather than true species, particularly in a young genus like *Entosphenus*. Paired *Lampetra planeri* and *Lampetra fluviatilis* can certainly hybridize resulting in substantial gene flow in parts of their range [60]. Our results indicate that incomplete lineage sorting may also play a role in the non-monophyly of some *Ichthyomyzon* species. Living most of their lives as larval ammocoetes burrowed in the substrate, lamprey biodiversity is likely under-described, and new species are still being discovered [61].

### (b) Antitropical distribution arose during extreme Late Cretaceous global warming

The hypothesis that lamprey antitropicality was driven by the rifting of Pangea relies on two key pieces of evidence: monophyly of Southern Hemisphere lampreys, which our dataset strongly supports, and an Early Jurassic divergence time for crown Petromyzontiformes. Our mean age estimate for crown Petromyzontiformes is firmly in the Cretaceous at 94.3 Ma, fully 70 million years younger than prior estimates in the Jurassic [6,62] (figure 3), though older than another recent estimate that did not obtain the monophyly of Southern Hemisphere families [14]. Major differences in our age estimates can be attributed to multiple factors, including the position of †*Mesomyzon* on the lamprey stem, and relying exclusively on mitochondrial DNA, which has been demonstrated to overestimate divergence times owing to saturation [22]. These younger divergence time estimates reject the role of Pangean rifting to explain the distribution of lampreys, as the northern and southern landmasses were well separated in the Late Cretaceous. Given our strong support for the monophyly of Southern Hemisphere lampreys, our data do not clearly support a recent hypothesis that lampreys dispersed out of the Southern Hemisphere during cooler climate windows [14].

Instead, this divergence time for crown lampreys coincides with extreme global warming during the Cenomanian–Turonian boundary event (CTBE; figure 3), the warmest period on Earth since the end-Permian extinction [58]. After a relatively cool period, temperatures climbed rapidly beginning around 107 Ma, with global average temperatures rising by over 7°C by the apex of the Cenomanian–Turonian Boundary Event at 93 Ma, not returning to previous levels until 75 Ma [58]. The CTBE resulted in major extinctions in marine invertebrates [63], large marine reptiles including ichthyosaurs and pliosaurs [64], and freshwater fishes [65]. Average tropical temperatures would have climbed above 34°C at the peak of the CTBE [58]. This exceeds the temperatures known to be lethal to ammocoetes [17] and likely would have made equatorial latitudes uninhabitable for larval lampreys. Vicariance due to rising palaeotropical temperatures has been hypothesized as a mechanism to explain the distributions of other antitropical temperate-adapted fishes [66,67]. We propose that inhospitable temperatures in the tropics during the CTBE drove vicariance between Northern and Southern Hemisphere lampreys. It is worth noting two caveats: first, with long, old branches our HPD interval is quite large at the crown lamprey node (figure 2*b*); second, lamprey fossils are rare and thus far have only been found as stem lineages, so the distribution of crown lampreys during this time is not known.

### (c) Diversification of extant lampreys in a cooling world

Extant lamprey lineages have thrived in the cooler periods of the Cenozoic. In the Southern Hemisphere, Geotriidae and Modaciidae split during the dramatic cooling associated with the Cretaceous–Palaeogene (K-Pg) extinction event (figure 2*b*), a much younger age than previous estimates in the Mesozoic era [2,6,68]. Rather than a steady accumulation of lamprey lineages over time, our results indicate that most extant lamprey diversity is due to an acceleration of the diversification of Petromyzontidae (figure 4*b*), the family that contains over 90% of extant species. Like a recent study with fewer loci and taxa [14], we also find a significantly young age for this clade (figure 3). Our analysis puts the origin of this clade at the Oligocene–Miocene boundary, rapidly diversifying during a period of significant global cooling (figure 3). Temperate flora and fauna expanded during this period [69]. Several other holarctic clades of fishes with high rates of diadromy evolved during the early Miocene, including *Alosa* (shads) [70,71], the salmonid genera *Onchorynchus*and *Salvenius* [72] and the Gasterosteidae (sticklebacks) [73]. It is possible the glacial activity during this period promoted diversification in diadromous clades [70,71]]. Alternatively, the rapid evolution of diverse life history strategies in Northern Hemisphere lampreys may have facilitated the increase in species diversity [74]. In contrast, the Southern Hemisphere families remain species-poor, and did not experience a shift in diversification during this period. While the Northern Hemisphere has large continental landmasses in temperate latitudes, the Southern Hemisphere is primarily dominated by open ocean—offering significantly less ecological opportunity for these families to diversify.

In addition to global cooling, major ocean gateways both closed and opened during the Miocene. The deepest split in Petromyzontidae separates an exclusively Atlantic-drainage clade (with one Caspian Sea endemic) separating *Caspiomyzon+Petromyzon+Ichthyomyzon* from all other genera, which contain both Pacific and Atlantic taxa. *Caspiomyzon* split from *Petromyzon+Ichthyomyzon* in the middle Miocene (15.5 Ma, HPD = 10.2–21.2 Ma), coinciding with the final closure of the Eastern Tethys Seaway, which separated the Atlantic from the Indian Ocean and isolated the Paratethys [75]. Subsequent reconnections in the Tortonian between the Mediterranean and the Eastern Paratethys [69] would explain the distribution of *Caspiomyzon* species in the Caspian Sea and the Mediterranean, the present-day Caspian Sea being a relic of the Eastern Paratethys. Primarily Pacific-drainage clades form the early branching lineages of the second major northern lamprey group, with multiple apparent dispersals back into the Atlantic. With several modern species inhabiting Arctic drainages, particularly in *Lethenteron*, dispersal via the Arctic Ocean seems a likely route for reaching the Atlantic.

## 5. Conclusions

Lampreys are a group of ancient jawless fishes that persist today, but our understanding of their evolutionary relationships and pattern of diversification has been limited owing to the lack of a comprehensive phylogeny. We estimated a well supported tree that resolves long-standing ambiguities in lamprey relationships and provides a framework for a revised generic classification of the group, sinking *Eudontomyzon* into the genus *Lampetra*. We estimated divergence dates using a total-evidence dating scheme with morphological data combined from two recent studies, the first analysis of this kind to use nuclear DNA. Our age estimates for the origin of crown Petromyzontiformes suggest a role for the extreme heat of the Cenomanian–Turonian Boundary Event causing vicariance and driving the disjunct antitropical distribution we see today. We also support a young Oligocene–Miocene boundary age for Northern Hemisphere lampreys, with a significant shift in diversification in this clade as the climate cooled in the Miocene.

## Data Availability

Raw sequence data can be accessed under NCBI Bioproject PRJNA1154577. Alignments, tree files, and code for exon-capture assembly have been made available on Dryad [76]. Supplementary material is available online [77].

## Dedication

We dedicate this paper to the late Philip Cochran, whose passion for lampreys lives on among his colleagues and friends.

## References

[1] Docker MF, Hume JB. 2015 Introduction: a surfeit of lampreys. In Lampreys: biology, conservation and control (ed. M Docker), pp. 1–26. Dordrecht, The Netherlands: Springer. (10.1007/978-94-017-9306-3_1)

[2] Miyashita T *et al*. 2019 Hagfish from the Cretaceous Tethys Sea and a reconciliation of the morphological–molecular conflict in early vertebrate phylogeny. Proc. Natl Acad. Sci. **116**, 2146–2151. (10.1073/pnas.1814794116) 30670644 PMC6369785

[3] Wilkie MP, Johnson NS, Docker MF. 2022 Invasive species control and management: the sea lamprey story. Fish Physiol. B. **39**, 489–579. (10.1016/bs.fp.2022.09.001)

[4] Maitland PS, Renaud CB, Quintella BR, Close DR, Docker MF. 2015 Conservation of native lampreys. In Lampreys: biology, conservation and control (ed. M Docker), pp. 375–428. Dordrecht, The Netherlands: Springer. (10.1007/978-94-017-9306-3_8)

[5] Lang NJ *et al*. 2009 Novel relationships among lampreys (Petromyzontiformes) revealed by a taxonomically comprehensive molecular data set. Am. Fish. Soc. Symp. **72**, 41–55. (10.47886/9781934874134.ch2)

[6] Brownstein CD, Near TJ. 2023 Phylogenetics and the Cenozoic radiation of lampreys. Curr. Biol. **33**, 397–404.(10.1016/j.cub.2022.12.018) 36586410

[7] Pereira AM *et al*. 2021 Putting European lampreys into perspective: a global‐scale multilocus phylogeny with a proposal for a generic structure of the Petromyzontidae. J. Zool. Syst. Evol. Res. **59**, 1982–1993. (10.1111/jzs.12522)

[8] Docker MF, Youson JH, Beamish RJ, Devlin RH. 1999 Phylogeny of the lamprey genus Lampetra inferred from mitochondrial cytochrome b and ND3 gene sequences. Can. J. Fish. Aquat. Sci. **56**, 2340–2349. (10.1139/f99-171)

[9] Gill HS, Renaud CB, Chapleau F, Mayden RL, Potter IC. 2003 Phylogeny of living parasitic lampreys (Petromyzontiformes) based on morphological data. Copeia **2003**, 687–703. (10.1643/IA02-085.1)

[10] Smith B, Walling A, Schwartz R. 2023 Phylogenomic investigation of lampreys (Petromyzontiformes). Mol. Phylogenet. Evol. **189**, 107942. (10.1016/j.ympev.2023.107942) 37804959

[11] Gess RW, Coates MI, Rubidge BS. 2006 A lamprey from the Devonian period of South Africa. Nature **443**, 981–984. (10.1038/nature05150) 17066033

[12] Janvier P, Lund R. 1983 Hardistiella montanensis n. gen. et sp. (Petromyzontida) from the lower Carboniferous of Montana, with remarks on the affinities of the lampreys. J. Vertebr. Paleontol. **2**, 407–413. (10.1080/02724634.1983.10011943)

[13] Lund R, Janvier P. 1986 A second lamprey from the Lower Carboniferous (Namurian) of Bear Gulch, Montana (USA). Geobios **19**, 647–652. (10.1016/S0016-6995(86)80061-4)

[14] Wu F, Janvier P, Zhang C. 2023 The rise of predation in Jurassic lampreys. Nat. Commun. **14**, 6652. (10.1038/s41467-023-42251-0) 37907522 PMC10618186

[15] Chang M, Zhang J, Miao D. 2006 A lamprey from the Cretaceous Jehol biota of China. Nature **441**, 972–974. (10.1038/nature04730) 16791193

[16] Clements T, Purnell M, Gabbott S. 2019 The Mazon Creek Lagerstätte: a diverse late Paleozoic ecosystem entombed within siderite concretions. J. Geol. Soc. **176**, 1–11. (10.1144/jgs2018-088)

[17] Potter IC, Beamish FWH. 1975 Lethal temperatures in ammocoetes of four species of lampreys. Acta Zool. **56**, 85–91. (10.1111/j.1463-6395.1975.tb00084.x)

[18] Ferreira AF, Quintella BR, Maia C, Mateus CS, Alexandre CM, Capinha C, Almeida PR. 2013 Influence of macrohabitat preferences on the distribution of European brook and river lampreys: implications for conservation and management. Biol. Conserv. **159**, 175–186. (10.1016/j.biocon.2012.11.013)

[19] Potter IC, Gill HS, Renaud CB, Haoucher D. 2015 The taxonomy, phylogeny, and distribution of lampreys. In Lampreys: biology, conservation and control (ed. M Docker), pp. 35–66. Dordrecht, The Netherlands: Springer. (10.1007/978-94-017-9306-3_2)

[20] Miyashita T, Gess RW, Tietjen K, Coates MI. 2021 Non-ammocoete larvae of Palaeozoic stem lampreys. Nature **591**, 408–412. (10.1038/s41586-021-03305-9) 33692547

[21] Ludt WB. 2021 Missing in the middle: a review of equatorially disjunct marine taxa. Front. Mar. Sci. **8**, 660984. (10.3389/fmars.2021.660984)

[22] Dornburg A, Townsend JP, Friedman M, Near TJ. 2014 Phylogenetic informativeness reconciles ray-finned fish molecular divergence times. BMC Evol. Biol. **14**, 169. (10.1186/s12862-014-0169-0) 25103329 PMC4236503

[23] Bravo GA *et al*. 2019 Embracing heterogeneity: coalescing the Tree of Life and the future of phylogenomics. PeerJ **7**, e6399. (10.7717/peerj.6399) 30783571 PMC6378093

[24] Fricke R, Eschmeyer WN, Laan R. 2023 Eschmeyer’s catalog of fishes: genera, species, references. See http://researcharchive.calacademy.org/research/ichthyology/catalog/fishcatmain.asp (accessed 15 December 2023).

[25] Lamb TD, Patel H, Chuah A, Natoli RC, Davies WIL, Hart NS, Collin SP, Hunt DM. 2016 Evolution of vertebrate phototransduction: cascade activation. Mol. Biol. Evol. **33**, 2064–2087. (10.1093/molbev/msw095) 27189541 PMC4948711

[26] Smith JJ *et al*. 2013 Sequencing of the sea lamprey (Petromyzon marinus) genome provides insights into vertebrate evolution. Nat. Genet. **45**, 415–421, (10.1038/ng.2568) 23435085 PMC3709584

[27] Smith JJ *et al*. 2018 The sea lamprey germline genome provides insights into programmed genome rearrangement and vertebrate evolution. Nat. Genet. **50**, 270–277. (10.1038/s41588-017-0036-1) 29358652 PMC5805609

[28] Eddy SR. 2011 Accelerated profile HMM searches. PLoS Comput. Biol. **7**, e1002195. (10.1371/journal.pcbi.1002195) 22039361 PMC3197634

[29] Hughes LC *et al*. 2018 Comprehensive phylogeny of ray-finned fishes (Actinopterygii) based on transcriptomic and genomic data. Proc. Natl Acad. Sci. **115**, 6249–6254. (10.1073/pnas.1719358115) 29760103 PMC6004478

[30] Hughes LC, Ortí G, Saad H, Li C, White WT, Baldwin CC, Crandall KA, Arcila D, Betancur-R R. 2021 Exon probe sets and bioinformatics pipelines for all levels of fish phylogenomics. Mol. Ecol. Resour. **21**, 816–833. (10.1111/1755-0998.13287) 33084200

[31] Bolger AM, Lohse M, Usadel B. 2014 Trimmomatic: a flexible trimmer for Illumina sequence data. Bioinformatics **30**, 2114–2120. (10.1093/bioinformatics/btu170) 24695404 PMC4103590

[32] Allen JM, LaFrance R, Folk RA, Johnson KP, Guralnick RP. 2018 aTRAM 2.0: an improved, flexible locus assembler for NGS data. Evol. Bioinform. **14**, 117693431877454. (10.1177/1176934318774546) PMC5987885 29881251

[33] Haas BJ *et al*. 2013 De novo transcript sequence reconstruction from RNA-seq using the Trinity platform for reference generation and analysis. Nat. Protoc. **8**, 1494–1512. (10.1038/nprot.2013.084) 23845962 PMC3875132

[34] Ranwez V, Douzery EJP, Cambon C, Chantret N, Delsuc F. 2018 MACSE v2: toolkit for the alignment of coding sequences accounting for frameshifts and stop codons. Mol. Biol. Evol. **35**, 2582–2584. (10.1093/molbev/msy159) 30165589 PMC6188553

[35] Arcila D *et al*. 2021 Testing the utility of alternative metrics of branch support to address the ancient evolutionary radiation of tunas, stromateoids, and allies (Teleostei: Pelagiaria). Syst. Biol. **70**, 1123–1144. (10.1093/sysbio/syab018) 33783539

[36] Simion P *et al*. 2017 A large and consistent phylogenomic dataset supports sponges as the sister group to all other animals. Curr. Biol. **27**, 958–967. (10.1016/j.cub.2017.02.031) 28318975

[37] Mai U, Mirarab S. 2018 TreeShrink: fast and accurate detection of outlier long branches in collections of phylogenetic trees. BMC Genom. **19**, 272. (10.1186/s12864-018-4620-2) PMC5998883 29745847

[38] Minh BQ, Schmidt HA, Chernomor O, Schrempf D, Woodhams MD, von Haeseler A, Lanfear R. 2020 IQ-TREE 2: new models and efficient methods for phylogenetic inference in the genomic era. Mol. Biol. Evol. **37**, 1530–1534. (10.1093/molbev/msaa015) 32011700 PMC7182206

[39] Chernomor O, von Haeseler A, Minh BQ. 2016 Terrace aware data structure for phylogenomic inference from supermatrices. Syst. Biol. **65**, 997–1008. (10.1093/sysbio/syw037) 27121966 PMC5066062

[40] Kalyaanamoorthy S, Minh BQ, Wong TKF, von Haeseler A, Jermiin LS. 2017 ModelFinder: fast model selection for accurate phylogenetic estimates. Nat. Methods **14**, 587–589. (10.1038/nmeth.4285) 28481363 PMC5453245

[41] Hoang DT, Chernomor O, von Haeseler A, Minh BQ, Vinh LS. 2018 UFBoot2: improving the ultrafast bootstrap approximation. Mol. Biol. Evol. **35**, 518–522. (10.1093/molbev/msx281) 29077904 PMC5850222

[42] Crotty SM, Minh BQ, Bean NG, Holland BR, Tuke J, Jermiin LS, Haeseler AV. 2020 GHOST: recovering historical signal from heterotachously evolved sequence alignments. Syst. Biol. **69**, 249–264. (10.1093/sysbio/syz051) 31364711

[43] Zhang C, Rabiee M, Sayyari E, Mirarab S. 2018 ASTRAL-III: polynomial time species tree reconstruction from partially resolved gene trees. BMC Bioinform. **19**, 153. (10.1186/s12859-018-2129-y) PMC5998893 29745866

[44] Minh BQ, Hahn MW, Lanfear R. 2020 New methods to calculate concordance factors for phylogenomic datasets. Mol. Biol. Evol. **37**, 2727–2733. (10.1093/molbev/msaa106) 32365179 PMC7475031

[45] Mo YK, Lanfear R, Hahn MW, Minh BQ. 2023 Updated site concordance factors minimize effects of homoplasy and taxon sampling. Bioinformatics **39**, btac741. (10.1093/bioinformatics/btac741) 36383168 PMC9805551

[46] Goloboff PA, Morales ME. 2023 TNT version 1.6, with a graphical interface for MacOS and Linux, including new routines in parallel. Cladistics **39**, 144–153. (10.1111/cla.12524) 36682054

[47] Bouckaert R, Heled J, Kühnert D, Vaughan T, Wu CH, Xie D, Suchard MA, Rambaut A, Drummond AJ. 2014 BEAST 2: a software platform for Bayesian evolutionary analysis. PLoS Comput. Biol. **10**, e1003537. (10.1371/journal.pcbi.1003537) 24722319 PMC3985171

[48] Heath TA, Huelsenbeck JP, Stadler T. 2014 The fossilized birth–death process for coherent calibration of divergence-time estimates. Proc. Natl Acad. Sci. **111**, E2957–E2966. (10.1073/pnas.1319091111) 25009181 PMC4115571

[49] Zhang C, Stadler T, Klopfstein S, Heath TA, Ronquist F. 2016 Total-evidence dating under the fossilized birth–death process. Syst. Biol. **65**, 228–249. (10.1093/sysbio/syv080) 26493827 PMC4748749

[50] Douglas J, Zhang R, Bouckaert R. 2021 Adaptive dating and fast proposals: revisiting the phylogenetic relaxed clock model. PLoS Comput. Biol. **17**, e1008322. (10.1371/journal.pcbi.1008322) 33529184 PMC7880504

[51] Benton MJ, Donoghue PCJ, Asher RJ, Friedman M, Near TJ, Vinther J. 2015 Constraints on the timescale of animal evolutionary history. Palaeontol. Electron. **2015**, 18.1.1FC. (10.26879/424)

[52] Paradis E, Schliep K. 2019 ape 5.0: An environment for modern phylogenetics and evolutionary analyses in R. Bioinformatics **35**, 526–528. (10.1093/bioinformatics/bty633) 30016406

[53] Revell LJ. 2012 phytools: An R package for phylogenetic comparative biology (and other things). Methods Ecol. Evol. **3**, 217–223. (10.1111/j.2041-210X.2011.00169.x)

[54] Pybus OG, Harvey PH. 2000 Testing macro-evolutionary models using incomplete molecular phylogenies. Proc. R. Soc. Lond. B **267**, 2267–2272. (10.1098/rspb.2000.1278) PMC1690817 11413642

[55] Rabosky DL, Grundler M, Anderson C, Title P, Shi JJ, Brown JW, Huang H, Larson JG. 2014 BAMMtools: An R package for the analysis of evolutionary dynamics on phylogenetic trees . Methods Ecol. Evol. **5**, 701–707. (10.1111/2041-210X.12199)

[56] Rabosky DL. 2014 Automatic detection of key innovations, rate shifts, and diversity-dependence on phylogenetic trees. PLoS One **9**, e89543. (10.1371/journal.pone.0089543) 24586858 PMC3935878

[57] Plummer M, Best N, Cowles K, Vines K. 2006 CODA: convergence diagnosis and output analysis for MCMC. R. News **6**, 7–11. https://journal.r-project.org/articles/RN-2006-002/

[58] Scotese CR, Song H, Mills BJW, van der Meer DG. 2021 Phanerozoic paleotemperatures: the Earth’s changing climate during the last 540 million years. Earth Sci. Rev. **215**, 103503. (10.1016/j.earscirev.2021.103503)

[59] Bailey RM. 1980 Comments on the classification and nomenclature of lampreys — an alternative view. Can. J. Fish. Aquat. Sci. **37**, 1626–1629. (10.1139/f80-209)

[60] Rougemont Q, Gagnaire PA, Perrier C, Genthon C, Besnard AL, Launey S, Evanno G. 2017 Inferring the demographic history underlying parallel genomic divergence among pairs of parasitic and nonparasitic lamprey ecotypes. Mol. Ecol. **26**, 142–162. (10.1111/mec.13664) 27105132

[61] Rüber L, Gandolfi A, Foresti D, Paltrinieri L, Splendiani A, Seehausen O. 2023 Phylogenetic and biogeographic history of brook lampreys (Lampetra: Petromyzontidae) in the river basins of the Adriatic Sea based on DNA barcode data. Ecol. Evol. **13**, e10496. (10.1002/ece3.10496) 37674653 PMC10477476

[62] Kuraku S, Kuratani S. 2006 Time scale for cyclostome evolution inferred with a phylogenetic diagnosis of hagfish and lamprey cDNA sequences. Zool. Sci. **23**, 1053–1064. (10.2108/zsj.23.1053) 17261918

[63] Kauffman EG *et al*. 1995 Global change leading to biodiversity crisis in a greenhouse world: the Cenomanian-Turonian (Cretaceous) mass extinction. In Effects of past global change on life, pp. 47–71. Washington, DC: National Academies Press.

[64] Fischer V, Bardet N, Benson RBJ, Arkhangelsky MS, Friedman M. 2016 Extinction of fish-shaped marine reptiles associated with reduced evolutionary rates and global environmental volatility. Nat. Commun. **7**, 10825. (10.1038/ncomms10825) 26953824 PMC4786747

[65] Eaton JG, Kirkland JI, Howard Hutchison J, Denton R, OʼNeill RC, Michael Parrish J. 1997 Nonmarine extinction across the Cenomanian-Turonian boundary, southwestern Utah, with a comparison to the Cretaceous-Tertiary extinction event. Geol. Soc. Am. Bull. **109**, 560–567. (10.1130/0016-7606(1997)1092.3.CO;2)

[66] Ludt WB, Myers CE. 2021 Distinguishing between dispersal and vicariance: a novel approach using anti‐tropical taxa across the fish Tree of Life. J. Biogeogr. **48**, 577–589. (10.1111/jbi.14021)

[67] White BN. 1986 The isthmian link, antitropicality and American biogeography: distributional history of the Atherinopsinae (Pisces: Atherinidae). Syst. Biol. **35**, 176–194. (10.1093/sysbio/35.2.176)

[68] Tims AR, Unmack PJ, Ho SYW. 2021 A fossil-calibrated time-tree of all Australian freshwater fishes. Mol. Phylogenet. Evol. **161**, 107180. (10.1016/j.ympev.2021.107180) 33887481

[69] Steinthorsdottir M *et al*. 2021 The Miocene: the future of the past. Paleoceanogr. Paleoclimatol. **36**, e2020PA004037. (10.1029/2020PA004037)

[70] Bloom DD, Lovejoy NR. 2014 The evolutionary origins of diadromy inferred from a time-calibrated phylogeny for Clupeiformes (herring and allies). Proc. R. Soc. B **281**, 20132081. (10.1098/rspb.2013.2081) PMC3906930 24430843

[71] Wang Q *et al*. 2022 Molecular phylogenetics of the Clupeiformes based on exon-capture data and a new classification of the order. Mol. Phylogenet. Evol. **175**, 107590. (10.1016/j.ympev.2022.107590) 35850406

[72] Lecaudey LA, Schliewen UK, Osinov AG, Taylor EB, Bernatchez L, Weiss SJ. 2018 Inferring phylogenetic structure, hybridization and divergence times within Salmoninae (Teleostei: Salmonidae) using RAD-sequencing. Mol. Phylogenet. Evol. **124**, 82–99. (10.1016/j.ympev.2018.02.022) 29477383

[73] Wang Y, Wang Y, Zhao Y, Kravchenko AY, Merilä J, Guo B. 2022 Phylogenomics of northeast Asian Pungitius sticklebacks . Divers. Distrib. **28**, 2610–2621. (10.1111/ddi.13423)

[74] Docker MF. 2009 A review of the evolution of nonparasitism in lampreys and an update of the paired species concept. Am. Fish. Soc. Symp. **72**, 71–114. (10.47886/9781934874134.ch4)

[75] Rögl F. 1999 Mediterranean and Paratethys. Facts and hypotheses of an Oligocene to Miocene paleogeography (short overview). Geol. Carpath. **50**, 339–349.

[76] Hughes LC, Bloom D, Piller K, Lang N, Mayden R. 2024 Data from: Phylogenomic resolution of lampreys reveals the recent evolution of an ancient vertebrate lineage. Dryad Digital Repository. (10.5061/dryad.qfttdz0s2) PMC11706654 39772957

[77] Hughes LC, Bloom DD, Piller K, Lang N, Mayden R. 2024 Supplementary material from: Phylogenomic resolution of lampreys reveals the recent evolution of an ancient vertebrate lineage. Figshare. (10.6084/m9.figshare.c.7571811) PMC11706654 39772957

